# The influence of fiber reinforcement on the physico-mechanical properties and microstructure of artificial stones with marble and quartzite waste aggregates

**DOI:** 10.1038/s41598-025-31710-x

**Published:** 2025-12-18

**Authors:** Ali Uromeihy, Hassan Shafaat-talab Dehghani, Mohammad Reza Nikudel, Zahra Sepahvand

**Affiliations:** 1 Present Address: Department of Engineering Geology, Faculty of Basic Science, Tarbiat Modares University, Jalal Al Ahmad Street, Tehran, Iran; 2 Department of Mining Engineering, Faculty of Technology and Engineering, Lorestan University, Lorestan, Iran; 3 Azarin Pars Artificial Stone Production Factory, Lorestan, Iran

**Keywords:** Artificial stone, Resin, Glass and polypropylene fibers, Marble, Quartzite, Flexural strength, Engineering, Materials science

## Abstract

This study fabricated artificial stone samples using marble (carbonate-based) and quartzite (siliceous-based) waste aggregates, their corresponding fine powders, and Unsaturated Polyester Resin (UPR) via the Vacuum, Vibration, and Compression (VVC) method. After evaluating three resin percentages for each aggregate, the optimal formulations- Artificial Marble with 10 wt% resin (AM-10R) and Artificial Quartzite with 12 wt% resin (AQ-12R) were reinforced with Polypropylene (PP) and Glass fibers (GF). Different results were observed despite identical processing conditions. Fiber reinforcement in artificial quartzite maintained compressive strength (up to + 5% relative to the control) and showed a trend toward improved flexural strength (up to + 12.6% in the optimal formulation). In contrast, artificial marble exhibited significant reductions in compressive strength (16.5–22.8%) and flexural strength (4–21%), primarily attributed to increased porosity from 2.75% to 5.83%. Laser particle size analysis attributed this divergence to the different powder characteristics. The marble powder was significantly finer due to marble’s lower hardness, resulting in a stiffer resin-powder mixture with reduced workability. This impeded efficient air removal during the VVC process. The addition of fibers further worsened this issue in the marble-based composite, leading to higher porosity and diminished reinforcement efficiency. Therefore, Effective fiber reinforcement for strength improvement depends on precise porosity control through optimized manufacturing parameters, which is highly dependent on the aggregate-powder system’s properties. These findings provide practical guidance for producing stronger, eco-friendly artificial stones from industrial waste using optimized fiber–resin systems.

**Supplementary Information:**

The online version contains supplementary material available at 10.1038/s41598-025-31710-x.

## Introduction

Natural stone is widely used in construction due to its accessibility, aesthetic appeal, durability, and sustainability. However, its extraction, transportation, and processing are often time-consuming and costly^1^. The overall recovery rate for construction stone production is estimated to be only about 29%, which highlights substantial resource loss and considerable waste generation^2^. To overcome these issues and offer a more affordable alternative, artificial or engineered stone has been developed^1^. These materials typically contain 70 to 95 wt % mineral particles embedded in a matrix, typically composed of polymer resins, cement, or ceramics^3^.

The production of artificial stone involves combining precisely graded natural stone fragments with a resin binder and other additives, with marble and quartz being the most common stone bases^4^. These additives, when used in small percentages, can modify the aesthetic appearance and improve the chemical and functional attributes of the artificial stone^4^.

Manufacturers of artificial stone normally use unsaturated polyester resin (UPR) as a binder for production due to its high-performance properties and low cost^4,5^. Research indicates that in UPR, Methyl Ethyl Ketone Peroxide (MEKP) is typically added at around 1wt% as a coupling agent, while cobalt is incorporated at 0.5-1wt% to act as a catalyst or accelerator^6,7^.

Synthetic fibers are also utilized as additives in resin and composite formulations^8^. Polymer composites are essentially multiphase materials where a polymer matrix is combined with fillers and reinforcing fibers. This combination results in a material with enhanced properties beyond those of its individual components. Fillers are often added to increase volume, lower costs, reduce density, or improve aesthetics, while fibers are incorporated to reinforce the polymer matrix, thereby increasing stiffness and strength^9,10^. Since the 1960s, advanced composite materials have been increasingly used across various engineering sectors, including aerospace, marine, and construction, due to their superior properties such as high strength-to-weight ratio, stiffness, and excellent resistance to fatigue, corrosion and superior long-term stability compared to cement-based systems^8,9,11^. In civil engineering, Fiber Reinforced Polymer (FRP) composites have become prominent in strengthening structural components such as beams, slabs, and bridge decks^9,12^. Their proven ability to enhance stiffness and load-bearing capacity has also encouraged their use in artificial stone composites to improve mechanical performance and durability.

Artificial stones in demanding applications such as high-traffic flooring, building facades, and large-format thin slabs are often subjected to dynamic, impact, and cyclic loads. Under such conditions, resistance to fatigue, impact, and crack propagation becomes essential, often exceeding the capabilities of unreinforced stone. This study therefore investigates the potential of fiber reinforcement to enhance mechanical strength, thereby potentially allowing for reduced slab thickness. This approach is particularly advantageous for high-rise structures where minimizing dead load is critical.

Various types of fibers are used for construction purposes, including steel fibers, polypropylene fibers, glass fibers, carbon fibers, aramid fibers, and natural fibers such as hemp and straw. Notably, glass fibers (GF) and Polypropylene (PP) fibers are highly effective micro-reinforcements, frequently used to improve concrete performance^13,14^.

The addition of GF or PP fiber can bridge microcracks in the matrix, distribute stress, and prevent crack propagation at crack tips^15^. These fibers effectively inhibit crack propagation, both during initial crack formation and subsequent growth within the matrix^15,16^.

Most research in the construction sector focuses on the effects of fibers on concrete, with limited studies on fiber-reinforced, resin-based artificial stone. However, research on fiber-reinforced polymer concrete, a material analogous to resin-based artificial stone, provides valuable relevant insights. Polymer concrete is increasingly used in construction for walls, bridges, and tunnels due to its high strength and durability^17^, and fiber reinforcement further enhances its performance^18^.

For example, Reis (2005) reported that adding 2% carbon fibers and 1% glass fibers to polymer concrete with 20% epoxy resin increased compressive strength by 16% and 8.7%, respectively^19^. Other researchers working with epoxy composites containing 20–25% resin found that while 1wt% PP or glass fibers marginally improved strength, increasing the fiber content to 1.5 wt% caused reduction due to impaired resin penetration and weakened interfacial bonding^20^.

Manufacturing methods significantly influence the structural properties of composites. Lee and Shin showed that vacuum processing reduces porosity in glass fiber and polyurethane composites from approximately 1.9% to 0.3–0.4%^21^. However, Elalaoui found that incorporating 1–2 wt % carbon or 1 wt% PP fibers in epoxy polymer concrete (13% resin) increased porosity (though less than in conventional concrete) due to compaction issues. This fiber reinforcement reduced compressive and flexural strength, likely due to weak fiber–matrix bonding and elevated porosity^22^, a finding supported by earlier studies^23,24^.

Since previous research has not investigated the effects of GF and PP fibers in resin-based artificial stones, this study examines the influence of marble and quartzite aggregates, along with these fibers, on the composite’s physico-mechanical properties. By comparing results with fiber-free samples and analyzing the reasons for strength changes, this research provides critical insights into utilizing fibers effectively in resin-based artificial stone formulations.

## Materials and methodology

### Raw materials

The mineral portion of the produced artificial stones consists of angular sand-sized aggregates and very fine stone powder, which was used as a filler to reduce resin consumption. Two different aggregate types were utilized in this research: marble waste (a carbonate aggregate, CaCO₃) and quartzite (a siliceous aggregate, SiO_2_).

Marble is carbonated metamorphic rock with chemical composition CaCO_3_ and quartzite, silica sedimentary sandstone with chemical composition SiO_2_.

Marble is a medium-strength calcite-based rock (Mohs 3), whereas quartzite, a highly resistant siliceous rock (Mohs 7), exhibits greater strength but higher brittleness^25^, and due to its high brittleness, it is unsuitable for producing large-dimension stone slabs. In contrast, artificial stone technology utilizing resin binders mitigates this brittleness, enabling the production of large slabs from quartzite waste.

**Fig. 1:**
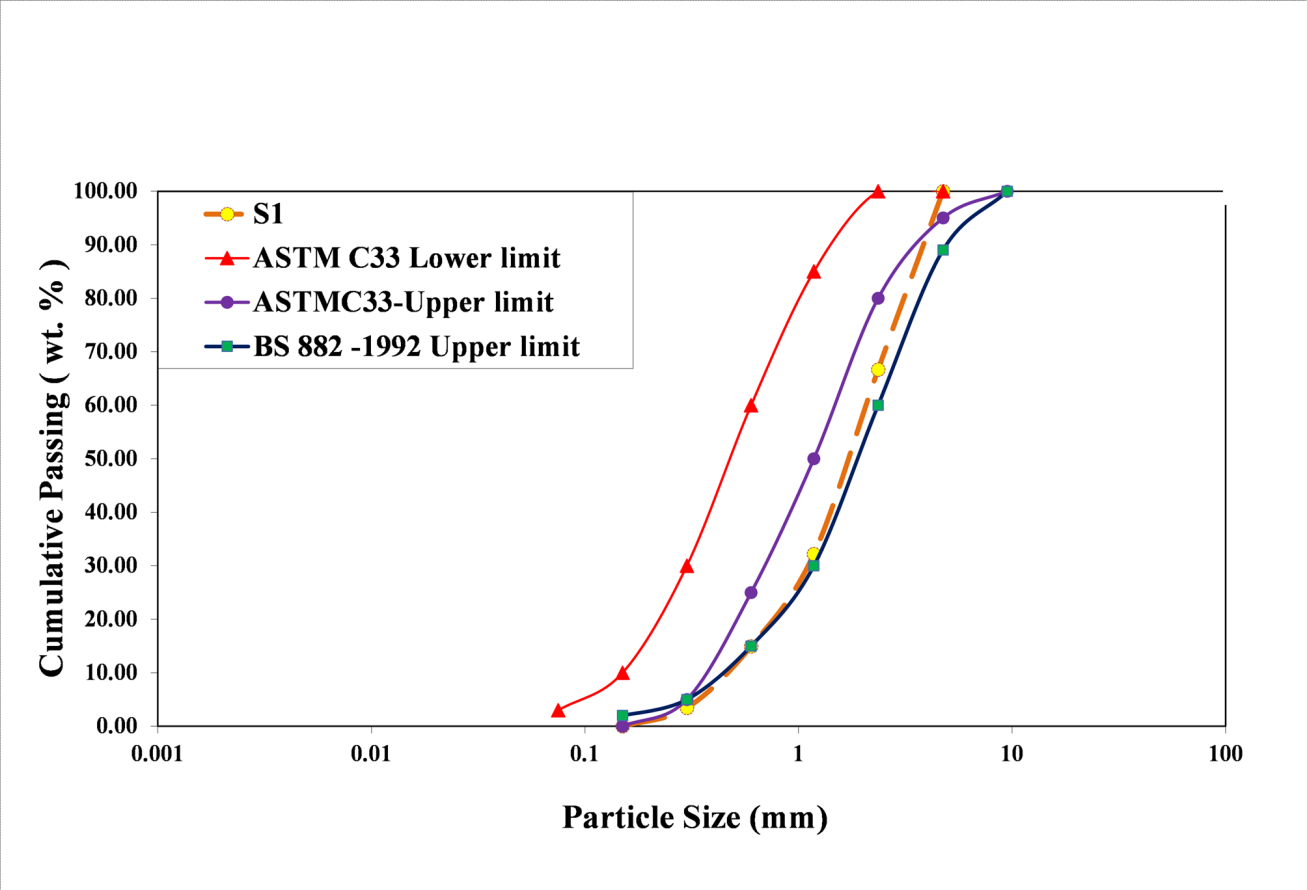
Particle size distribution of graded sand prepared from crushed marble and quartzite waste (S1, dashed line), compared to standard sand gradation limits (solid lines).

Through crushing and sieving of marble and quartzite waste, fine to coarse sand aggregates (0.15–4.75 mm) were prepared. The particle size distribution (Table 1, Curve S1 in Fig. 1) falls within the upper limit range specified by ASTM C33^26^ and BS 882:1992^27^standards for concrete sand.

Shishegaran et al.^28^, Labaran et al.^29^ and Osfouri et al.^30^ used the gradation curve defined for sand by ASTM C33 in their research as well.

The fine silicate and carbonate powders (derived from grinding quartzite and marble) passed through a No. 200 sieve (≤ 0.075 mm) and were used as fillers. These powders significantly reduced resin consumption by filling voids between aggregates, enhancing production cost-efficiency.

An orthophthalic UPR was employed as the binder. Methyl ethyl ketone peroxide (MEKP) as hardener and Silane as coupling agent. Table 2 summarizes the technical specifications of the employed resin.

Two types of fibers, GF and PP fibers, were used in this study (Table 3). Given the interior applications of the artificial stone (e.g., cladding and flooring), aesthetic compatibility was essential. Both PP and GF fibers are colorless and transparent, ensuring no adverse visual effects when incorporated appropriately.

**Table 1:** Particle size distribution of manufactured sand (aggregates) from crushed and sieved quartzite and marble waste.

Mesh no. of sieves	Grain size (mm)	Weight% of particles (wt %)
#4 to #8	2.36–4.75	33.5
#8 to #16	1.18–2.36	34.0
#16 to #30	0.6–1.18	17.5
#30 to #50	0.3–0.6	10.0
#50 to #100	0.15–0.3	5.0

**Table 2:** Technical specifications of the orthophthalic unsaturated polyester resin.

Properties	Unit	Value
Solid content	wt%	67 ± 2
Viscosity	mPa s (cps), (at 25 °C)	450 ± 100
Acid value	mgr KOH/ g Resin	35 ± 5
Density	g/cm^3^	1.12–1.15
Gel time	min, (at 25 °C)	10–15
Curing time	min, (at 25 °C)	15–20
Peak exothermic temperature	⁰C, (at 25 °C)	140–150
Flexural strength	MPa	100–110
Elongation at break	%	4–8

**Table 3:** Technical specifications of GF and PP fibers.

Property	GF	PP	Remarks
Tensile strength (MPa)	1400 ± 100	625–670 ± 25	Glass fibers show superior strength
Elastic modulus (GPa)	52 ± 3	5 ± 0.25	Glass fibers are more rigid
Elongation at break (%)	2.7 ± 0.2	14 ± 0.75	PP fibers offer greater flexibility
Density (g/cm^3^)	2.6	0.91	PP fibers are significantly lighter
Length (mm)	6	6	–
Fiber diameter (mm)	0.02	0.02–0.03	–
Melting point (°C)	400	170–180	Glass fibers have higher thermal stability

### Sample production method

In this investigation, three AQs and three AMs specimens were initially fabricated. Each aggregate type was combined with its corresponding fine powder (acting as filler) and resin using three different weight ratios. The optimal sample from each category was selected based on superior physico-mechanical properties, specifically lower porosity, higher mechanical strength, and minimal resin consumption. Subsequently, fibers were introduced into these optimized mixtures, and the outcomes were compared to fiber-free reference samples. Table 4 summarizes the mixture designs for all fiber-free samples.

As detailed in Table 4, the artificial marble samples were produced with resin contents of 8%, 10%, and 12% wt%, whereas the artificial quartzite samples utilized resin proportions of 10%, 12%, and 14%. Previous experiences show that UPR is usually used between 10% and 20% for artificial stone to be cost-effective and for workability and mobility^31^.

**Table 4:** Weight% of Raw materials used to production AMs and AQs.

	Sample no.	Graded sand aggregates (wt %)	Filler (wt %)	UP resin (wt %)
Artificial marble	AM-08	67	25	8
	AM-10	67	23	10
	AM-12	67	21	12
Artificial quartzite	AQ-10	63	27	10
	AQ-12	63	25	12
	AQ-14	63	23	14

For fiber-free samples, aggregates and filler powder were dry-mixed for 5 min. The resin was then blended with 1 wt% MEKP hardener and 1 wt% Silane coupling agent (2 min), followed by 5 min of mixing with the aggregates. The mixture was cast into a 250 × 250 × 50 mm^3^ steel mold, placed under vacuum (450 mmHg for 4 min), compacted (0.5 MPa for 5 min), and vibrated (50 Hz for 4 min) while maintaining both vacuum and compaction pressure. Finally, the samples were cured at 100 °C for 90 min.

This manufacturing method, which involves the simultaneous application of Vacuum, Vibration, and Compression pressure (VVC), is an established technique in artificial stone production^4^ and has been employed by various researchers^7,32–36^. The VVC process and the UPR were intentionally kept constant throughout all experiments to ensure that the effects of aggregate type (marble or quartzite) and fiber type (GF and PP) could be compared under identical processing conditions.

**Fig. 2:**
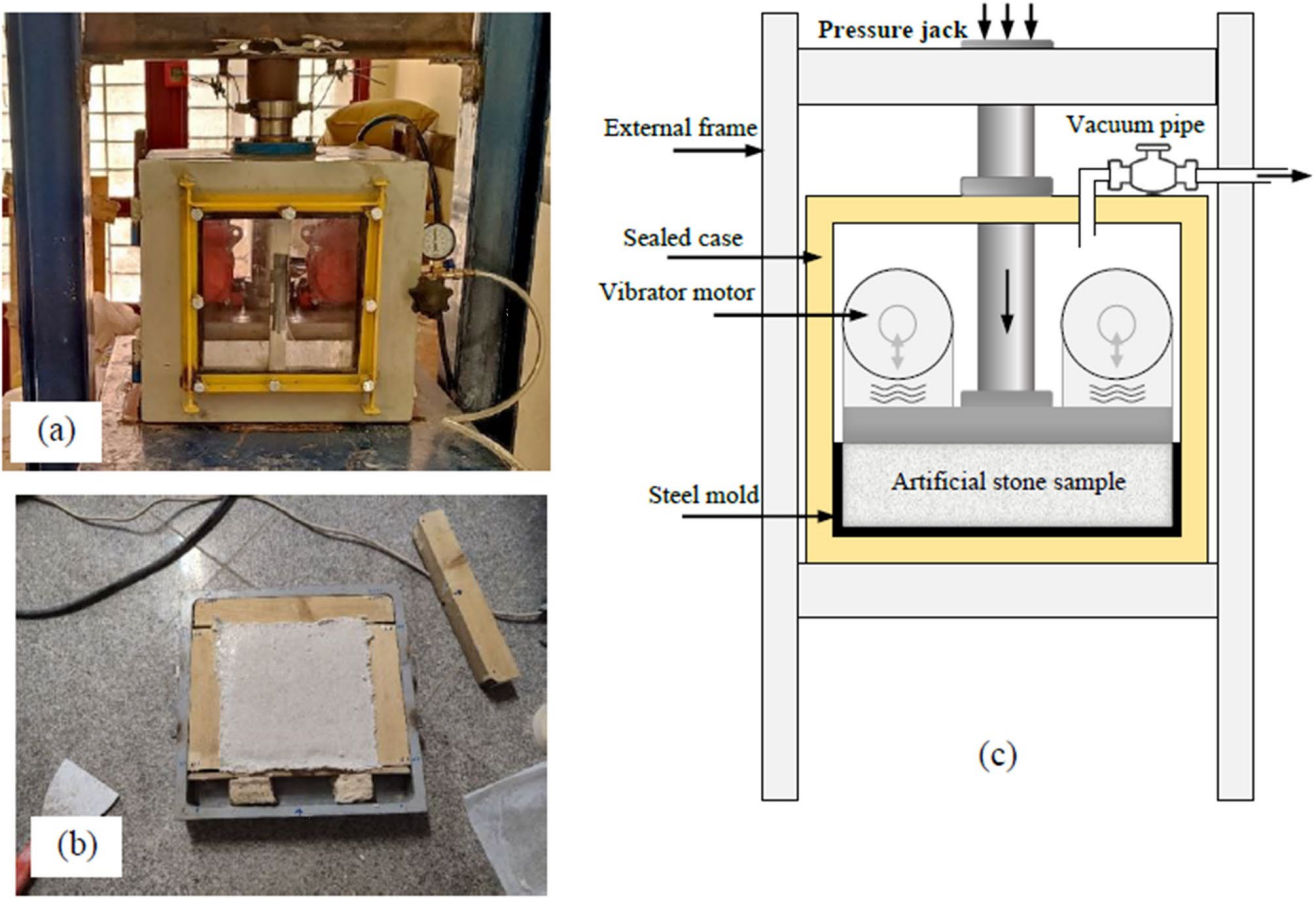
(**a**) General view of the laboratory vacuum–vibration–compression (VVC) machine, (**b**) steel mold and a produced artificial stone sample, and (**c**) schematic illustration of the VVC assembly used for specimen fabrication.

It should be noted that in this study, the VVC machine was custom-designed and fabricated on a laboratory scale by the research team based on technical investigation and industrial experiences (Fig. 2a–c).

**Fig. 3:**
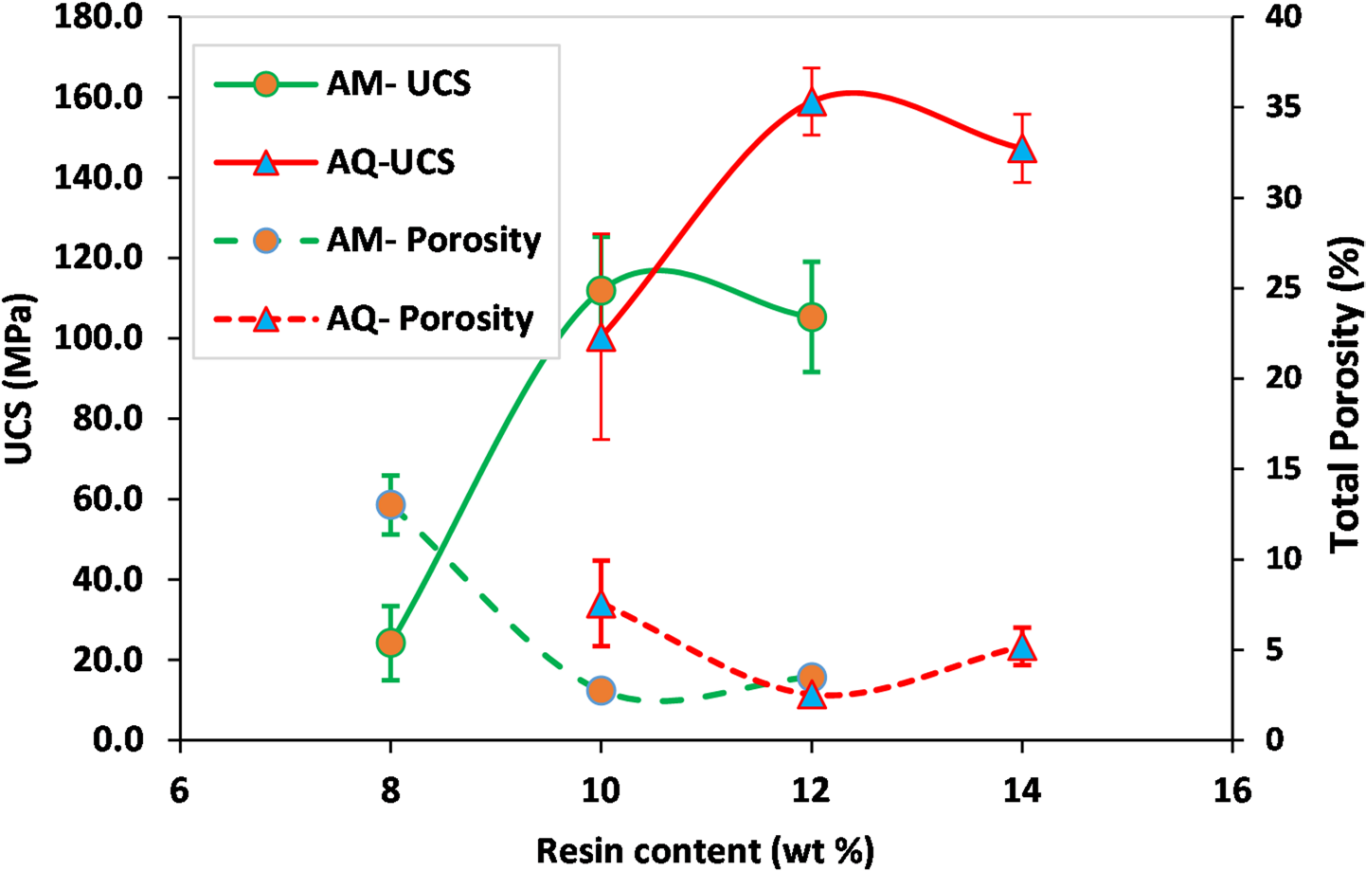
Uniaxial compressive strength (UCS) and total porosity of artificial marble (AM) and artificial quartzite (AQ) samples prepared with different resin contents.

**Fig. 4:**
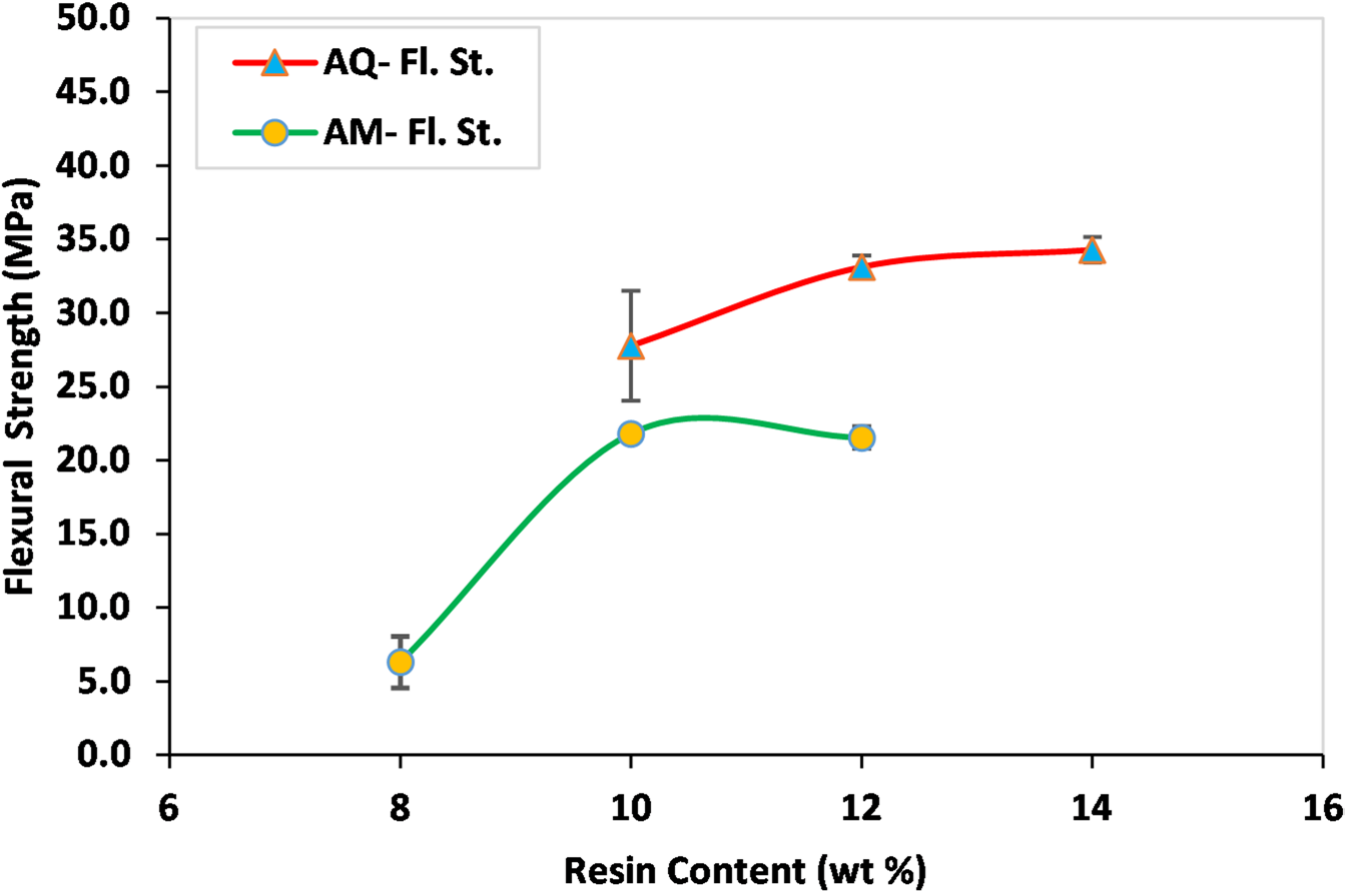
Flexural strength of AM and AQ samples prepared with different resin contents.

Following the fabrication and testing of the samples, the AM-10R (with 10% resin) from the AM group and the AQ-12R (with 12% resin) from the quartzite group were identified as exhibiting the best physico-mechanical properties. The most important parameters influencing the selection of the best fiber-free samples included the total porosity, Uniaxial Compressive Strength (UCS), and flexural strength. The results of these tests are presented in the form of charts in Figs. 3 and 4. The standards used for sample preparation and dimensions, as well as the methodology for each test, are provided in the following Section “Characterization of artificial stones”.

Based on the experimental results (Fig. 3), samples AM-8R and AQ-10R exhibited the highest porosity and lowest UCS in their respective groups. The insufficient resin content in these specimens failed to adequately fill the voids between mineral particles, making them unsuitable for further consideration.

The higher standard deviation observed in samples with lower resin content (AM-8R and AQ-10R) can be attributed to insufficient binder material. This deficiency likely leads to non-uniform distribution of resin among aggregate particles, resulting in a more heterogeneous microstructure and inconsistent mechanical performance across the tested specimens.

Among the AM series, AM-10R demonstrated the highest UCS (111.9 MPa) and lowest porosity (2.75%), while in the AQ series, AQ-12R showed the highest UCS (158.9 MPa) and lowest porosity (2.53%).

Based on ASTM C503, the minimum UCS for calcite marble must exceed 52 MPa^37^, while ASTM C615 specifies a minimum UCS of approximately 131 MPa for granites^38^. The results demonstrate that the AM-10R meets the ASTM requirements, and AQ-12R even achieves values comparable to hard igneous rocks such as granite. Through proper mixing of aggregates and stone powder with resin, and by employing vacuum, compression pressure, and vibration, high-strength artificial stones have been produced with both types of aggregates. It is worth noting that the average UCS of natural marble and natural quartzite in this study was measured at 102.3 MPa and 169.4 MPa, respectively. Thus, according to the results, it is evident that using stronger and harder aggregates enables the production of artificial stones with higher strength.

According to Fig. 4, both AM-10R and AQ-12R exhibit adequate flexural strength, demonstrating values of 21.8 MPa and 33.1 MPa, respectively. Based on ASTM C503, the minimum expected flexural strength for calcite marble exceeds 7 MPa. Furthermore, ASTM C615 specifies a minimum flexural strength of approximately 8.27 MPa for hard granites.

Excessive resin content (AM-12R: 12%, AQ-14R: 14%) resulted in slightly increased porosity and reduced compressive strength.

The study by Peng and Qin^7^ on fabricating artificial stone slabs from silica crucible waste and quartz sand, bonded with 8–12% UPR, yielded critical insights into the relationship between resin content and mechanical properties. These findings are consistent with the results of the present study, which also identified a distinct threshold resin content necessary for achieving optimal strength. At the minimum resin content of 8%, inadequate coverage left fine particles exposed, which substantially compromised both compressive and flexural strength. Their results indicated a sharp increase in mechanical properties as the resin content rose from 8% to 10%. Beyond 10%, the strength gains diminished, forming a near-plateau. The optimal performance was observed at 11% UPR, achieving peak strength values of 176.4 MPa in compression and 75.0 MPa in flexure. This optimum is attributed to the resin nearly completely filling the voids between SiO_2_ particles. A gradual decline in strength was noted beyond 11% UPR, likely resulting from the accumulation of excess resin within the inter-particle spaces, which can create stress concentration points. To justify this issue, it can be stated that when the resin content exceeds the amount required to fill the void spaces, the compaction force (applied during sample fabrication) is not effectively transferred to the aggregates. Additionally, due to the accumulation of resin, a fluid with high viscosity and adhesion properties, the likelihood of entrapped air bubbles increases.

In the main experimental stage of this study, GF and PP fibers were added in varying proportions to the previously identified best formulations (AM-10R and AQ-12R).

To evaluate the effect of fibers on the mechanical properties of artificial stone, GF and PP fibers were incorporated in the following formulations:

- GF-reinforced samples:
  Fiber length: 6 mm, volume fractions: 0.5%, 1%, and 1.5%, Includes: 3 artificial marble and 3 artificial quartzite samples with GF.
- PP fiber-reinforced samples:
  Fiber length: 6 mm, volume fractions: 0.25%, 0.5%, and 0.75%, Includes: 3 artificial marble and 3 artificial quartzite samples with PP fibers.
- Hybrid fiber-reinforced samples:
  Combination of 0.25% PP fibers and 1% GF (by volume), includes: 1 artificial marble and 1 artificial quartzite sample.

The mechanical and physical properties of these fiber-reinforced composites were then systematically evaluated against their fiber-free counterparts.

Fiber dosages were selected based on literature ranges for fiber-reinforced polymer composites.

A study on hybrid reinforced polypropylene fiber concrete (Hybrid-PFRC) beams found that an optimal polypropylene (PP) volume fraction of 0.4% yields the highest flexural strength, while 0.7% provides the best flexural toughness; beyond 0.4%, flexural strength decreases^39^.

Labaran et al. investigated the effect of fibers on the strength and durability enhancement of high-strength concrete (HSC)^29^. They used three types of fibers, GF, PP, and Polyvinyl Alcohol Fiber (PVAF), each 12 mm in length. PP fibers were incorporated at 0.25%, 0.5%, and 0.75% wt %, while GF were used at 0.5%, 1%, and 1.5% wt%. The sand particle grading followed ASTM C33.

Their results showed that with 1.5% GF, higher compressive, flexural, and tensile strengths were achieved compared to fiber-free samples. However, other fiber-reinforced samples exhibited lower strengths than plain concrete. A cost analysis of the samples revealed that those with polyvinyl alcohol fibers incurred the highest production costs. Even at 0.25% content, the use of PVAF increased production costs by approximately 50% compared to conventional concrete. In contrast, PP and GF, at their highest dosages, increased costs by 18% and 24%, respectively. This cost increase is significantly lower than that associated with PVAF.

In this research to precisely determine the advantages and disadvantages of using fibers in the artificial stone, the same UPR resin and manufacturing parameters (vacuum, compression pressure, and vibration) as those used for the fiber-free samples were employed.

### Characterization of artificial stones

- The determination of apparent density, water absorption was conducted according to ASTM C97^40^ and apparent porosity was according to International Society for Rock Mechanics (ISRM) using saturation and buoyancy techniques^41^.
- As a supplementary investigation, to determine total porosity, first, artificial stone samples were milled, and then the specific gravity (Gs) of each sample was calculated according to ASTM D 854^42^. Next the total porosity of the samples was calculated according to Eq. (1):

1 $${\mathrm{n}}\% = 1 - \frac{{\gamma _{{d~ \times ~100}} }}{{(G_{S} \times \gamma _{w} )}}$$

where n%: Total porosity; G_S_: Specific gravity; γ_d_: Apparent dry density of artificial and natural stone (g/cm^3^); γ_w_: Water density (at 4 °C).

Previous studies have only measured and compared the apparent porosity of artificial stones with natural stones, usually reporting lower values for artificial specimens^32,35,43–47^. In fact, the resin content in artificial stone prevents water penetration (during saturation-based porosity testing), leading to low apparent porosity. However, entrapped air bubbles in the polymer matrix may cause total porosity (n %) to significantly exceed apparent porosity.

While low apparent porosity benefits chemical resistance by limiting solvent penetration, total porosity remains critical as it degrades mechanical properties through pore-induced stress concentrations that promote crack initiation and propagation^48^.

**Fig. 5:**
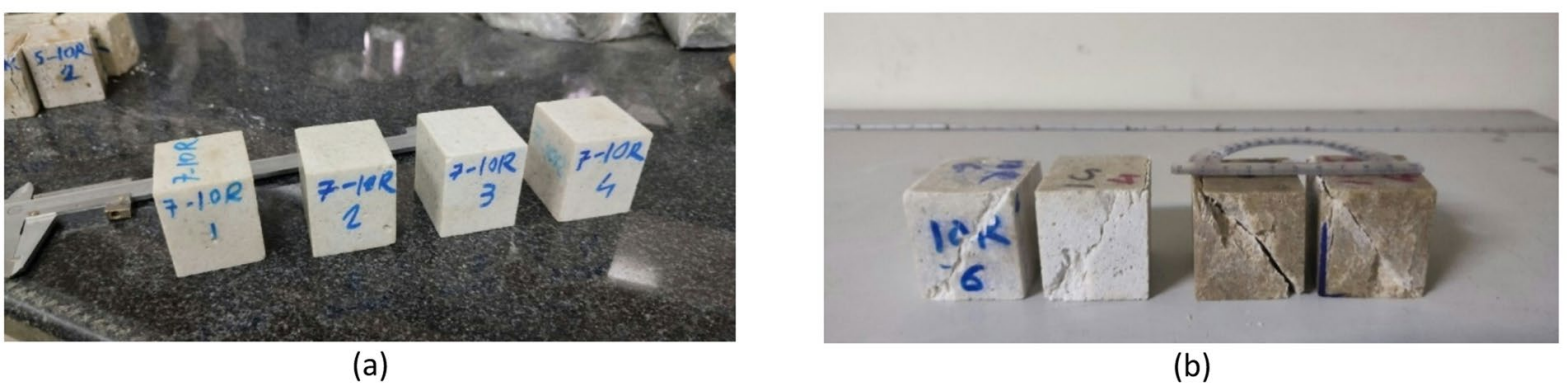
Typical cubic samples for the UCS test: (**a**) Artificial marbles before loading, (**b**) Artificial quartzites and marbles after failure.

**Fig. 6:**
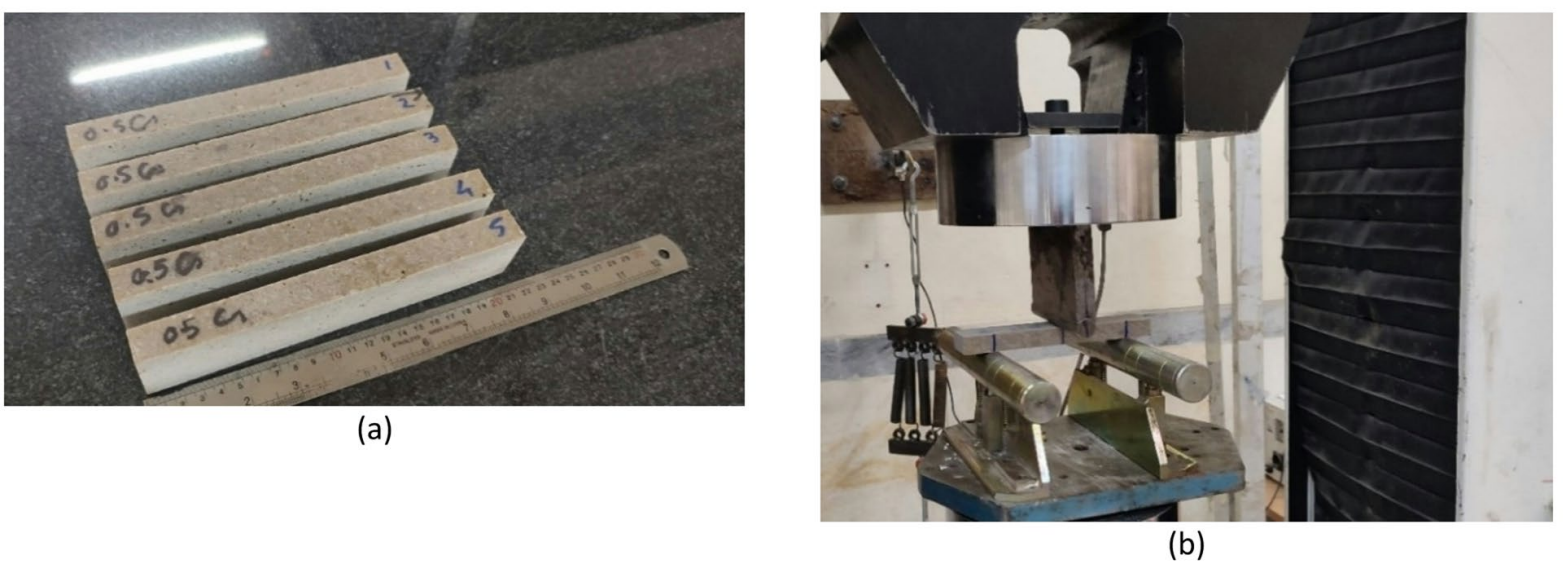
(**a**) Typical artificial quartzite specimens prepared for the flexural strength test, and (**b**) loading setup of the three-point bending test (mid-point loading).

- The UCS test was performed according to ASTM –C170, using 5 dried cubic samples (same as Fig. 5) with dimensions 50 ± 5 mm^49^.
- The microstructure observation was performed using a ZEISS scanning electron microscope (SEM) operating at 25 kV on gold-plated samples.
- The flexural strength (σ_f_) test was performed according to ASTM –D790^50^, using 5 samples with dimensions 18 × 40 × 200 mm(same as Fig. 6). The bending strength of the rectangular sample was calculated according to Eq. (2).

2 $$\sigma _{f} = \frac{{3PL}}{{2bd^{2} }}$$

where σ_f_ is the flexural strength (MPa), *P* is the maximum load when the specimen breaks (*N*), *L* is the span of the specimen (mm), *b* is the width of the specimen (mm), and *d* is the thickness of the specimen (mm).

## Results

The physico-mechanical test results for the fiber-reinforced samples, along with two selected fiber-free samples (AM-10R and AQ-12R), are presented in Table 5. Fiber-free results are highlighted for comparison. In nominating these samples, “PP” denotes polypropylene fibers, “GF” denotes glass fibers, and the number preceding them indicates the volumetric percentage of the fiber dosage.

**Table 5:** Results of physico-mechanical tests on fiber-added artificial stone samples and their comparison with plain samples (AM-10R and AQ-12R).

	Type of samples	Density	Water absorption	Apparent porosity	Total porosity	UCS	Flexural St.
		g/cm^3^	%	%	%	MPa	MPa
Artificial marbles	AM-10R	**2.40**	**0.12**	**0.29**	**2.75**	**111.9 ± 13.4**	**21.8 ± 0.5**
	AM-0.25PP	2.35	0.17	0.40	4.94	91.3 ± 5.4	20 ± 0.9
	AM-0.5PP	2.35	0.18	0.42	4.71	89.9 ± 7.3	21 ± 1.3
	AM-0.75PP	2.34	0.21	0.49	4.61	93.4 ± 12.7	20.9 ± 1.5
	AM-0.5G	2.34	0.17	0.41	5.30	90.6 ± 8.2	17.2 ± 1.6
	AM-1G	2.36	0.17	0.39	4.99	93.4 ± 10.2	18.3 ± 2.7
	AM-1.5G	2.33	0.28	0.66	5.57	87.2 ± 11.7	20 ± 1.1
	AM-0.25PP/1G	2.32	0.24	0.55	5.83	86.4 ± 5.1	18.6 ± 2
Artificial quartzites	AQ-12R	**2.31**	**0.16**	**0.36**	**2.53**	**158.9 ± 8.3**	**33.1 ± 0.8**
	AQZ-0.25PP	2.29	0.15	0.33	2.63	166.1 ± 20.9	37 ± 1.2
	AQZ-0.5PP	2.30	0.16	0.37	1.89	166.9 ± 15	36 ± 2.3
	AQZ-0.75PP	2.29	0.18	0.41	2.03	160.3 ± 20	35.7 ± 3
	AQZ-0.5G	2.29	0.14	0.31	3.54	155.5 ± 18.6	34.4 ± 3.3
	AQZ-1G	2.30	0.13	0.3	2.84	164.1 ± 19.4	36.6 ± 1.8
	AQZ-1.5G	2.29	0.13	0.31	2.80	163.8 ± 12.9	35.8 ± 1.7
	AQZ-0.25PP/1G	2.28	0.2	0.46	3.36	159.6 ± 13.9	35.9 ± 3.2

Fiber-free (plain) results are in bold for comparison.

The requisite charts were prepared to evaluate the impact of (PP) and (GF) dosage on the physical, mechanical properties of artificial stones.

### Physical properties

**Fig. 7:**
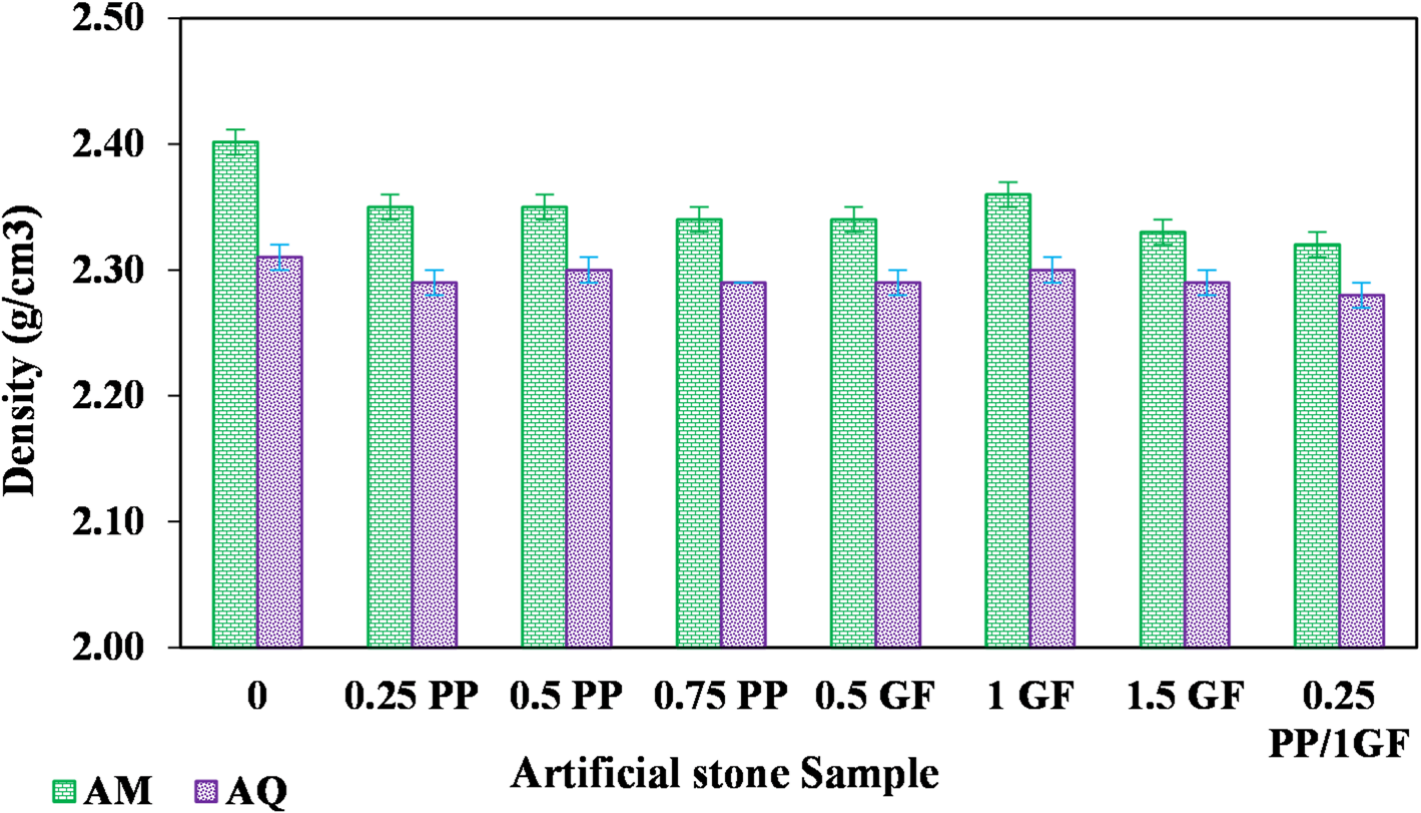
Variation in density of AM and AQ samples reinforced with different volume fractions of glass fiber (GF) and polypropylene (PP) fibers.

**Fig. 8:**
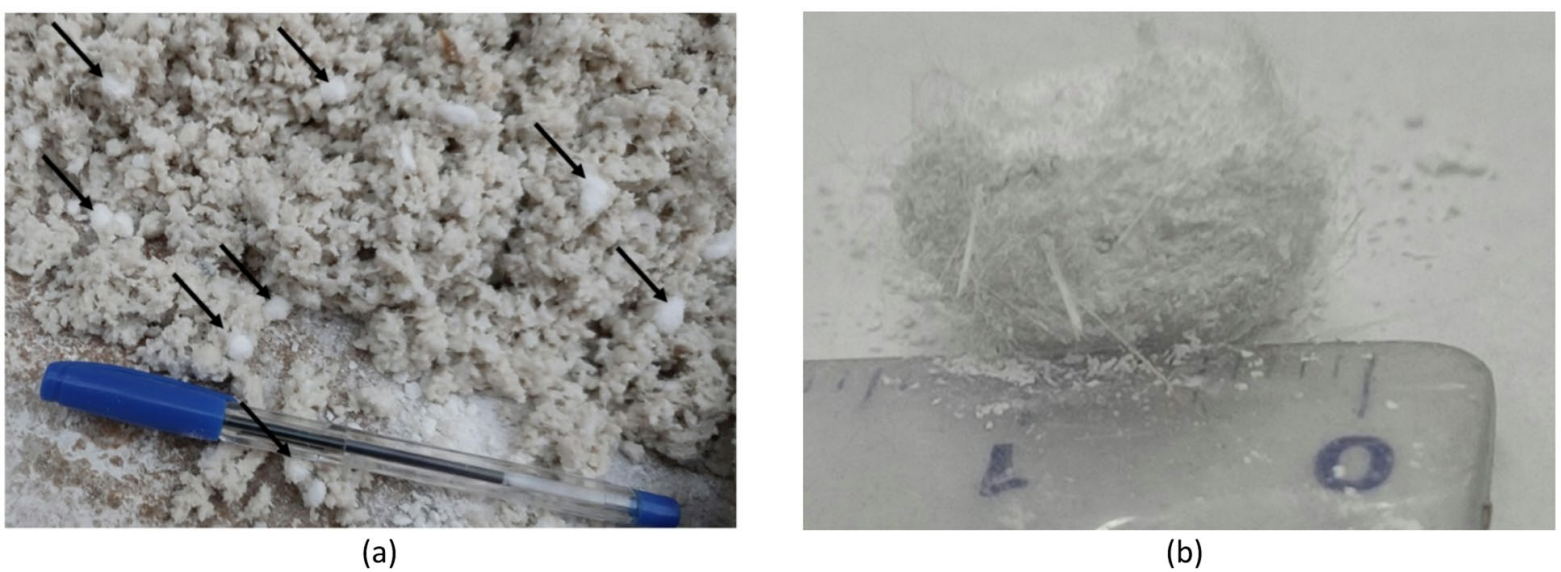
(**a**) Pronounced fiber agglomeration observed in the hybrid mixture containing 0.25% PP and 1% GF, and (**b**) close-up view showing interwoven glass (GF) and polypropylene (PP) fibers forming localized clusters.

The density variation of artificial stone samples with GF and PP fibers is shown in Fig. 7. Fiber addition caused a significant density reduction in AM samples (from 2.4 g/cm^3^ to 2.32–2.36 g/cm^3^), while the decrease was less pronounced in AQ samples. The maximum reduction occurred with hybrid PP/GF fibers, primarily due to fiber agglomeration and poor distribution, as illustrated in Fig. 8.

Mixing fibers with aggregates and stone powder in the dry state (before resin addition) must be performed gently to ensure uniform distribution and prevent agglomeration. Special attention is required for PP fibers due to their lower density compared to mineral aggregates.

In this study, the density of the natural marble and quartzite, from which the aggregates were derived, was measured and found to be 2.7 g/cm^3^ and 2.57 g/cm^3^, respectively, while the density of PP is 0.91 g/cm^3^. Marble is composed of calcite minerals, while quartzite is formed from quartz grains. The specific gravity of calcite is approximately 2.7, and that of quartz is about 2.65^51^. The lower specific gravity of quartz compared to calcite justifies the lower density of quartzite relative to marble.

High mixing speeds cause segregation of these lightweight fibers and promote agglomeration. Moreover, higher dosages further increase the risk of PP fiber segregation, as explicitly observed at approximately 0.75% dosage where agglomeration and separation clearly occurred. In contrast, GF are more easily mixed due to their comparable density with mineral particles, resulting in negligible segregation tendency.

Yuan et al. studied concrete with GF and PP fibers (0.45–1.35 vol%). PP fibers reduced UCS due to increased porosity and reduced homogeneity, while GF showed better performance with lower water absorption and higher compressive, flexural, and tensile strengths^14^.

**Fig. 9:**
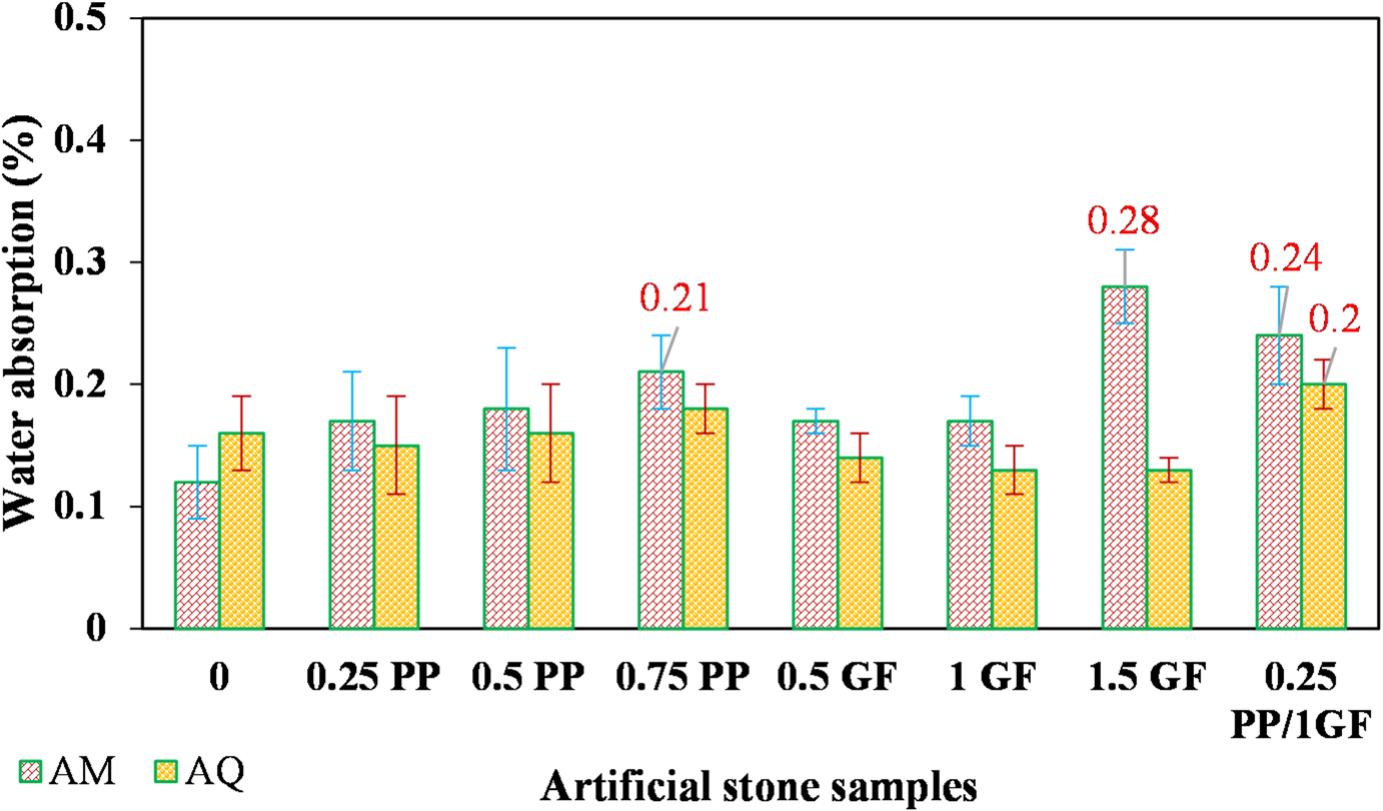
Variation of water absorption in AM and AQ samples with varying contents of GF and PP fibers.

**Fig. 10:**
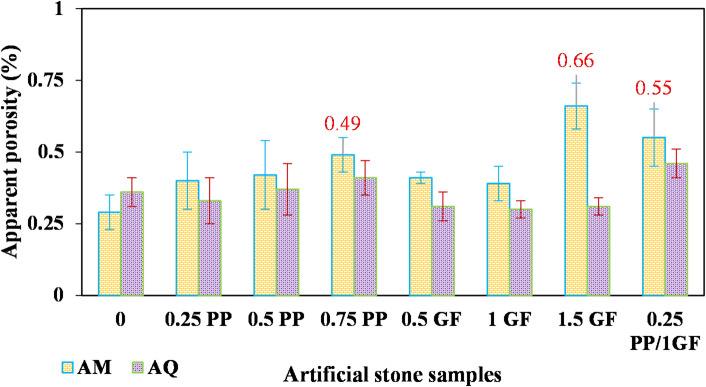
Variation of apparent porosity (%) in AM and AQ samples with varying contents of GF and PP fibers.

According to Fig. 9, the use of PP fibers has led to a gradual increase in water absorption in AM samples compared to the fiber-free sample. Water absorption in the three AM samples with 0.75% PP, 1.5% GF, and hybrid fibers exceeded the standard limit of 0.2% (ASTM-C 503). As shown in Fig. 10, their apparent porosity is also about 0.5% or higher. According to the recommendations provided by^52^, the apparent porosity of stones used for surface cladding should be less than 0.5%.

The addition of fibers had minimal impact on the water absorption and apparent porosity of the AQ samples. Except for the hybrid fiber specimen, all reinforced AQ samples exhibited values similar to or even below those of the fiber-free sample.

**Fig. 11:**
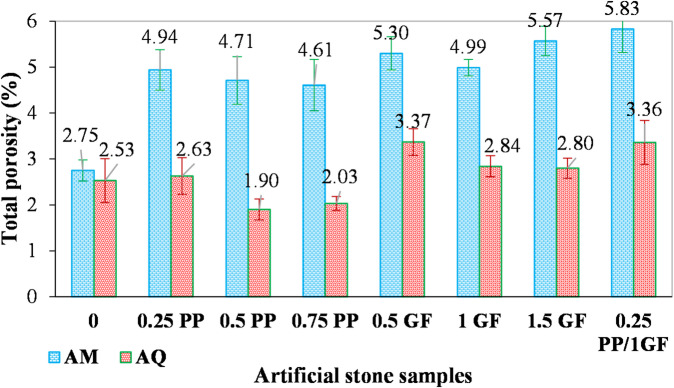
Variation of total porosity (%) in AM and AQ samples with varying contents of GF and PP fibers.

The total porosity of all fiber-reinforced samples (representing actual void volume) was calculated after specific gravity (Gs) measurement, with results shown in Fig. 11. The incorporation of fibers increased the total porosity in all AM samples, rising from 2.75% (for fiber-free) to a range of 4.61–5.83%, which consequently degraded the stone’s quality. In contrast, the total porosity of AQ samples changed minimally, with values ranging from 1.9% to 3.36% for reinforced specimens, compared to 2.53% for the fiber-free sample.

### Mechanical properties

**Fig. 12:**
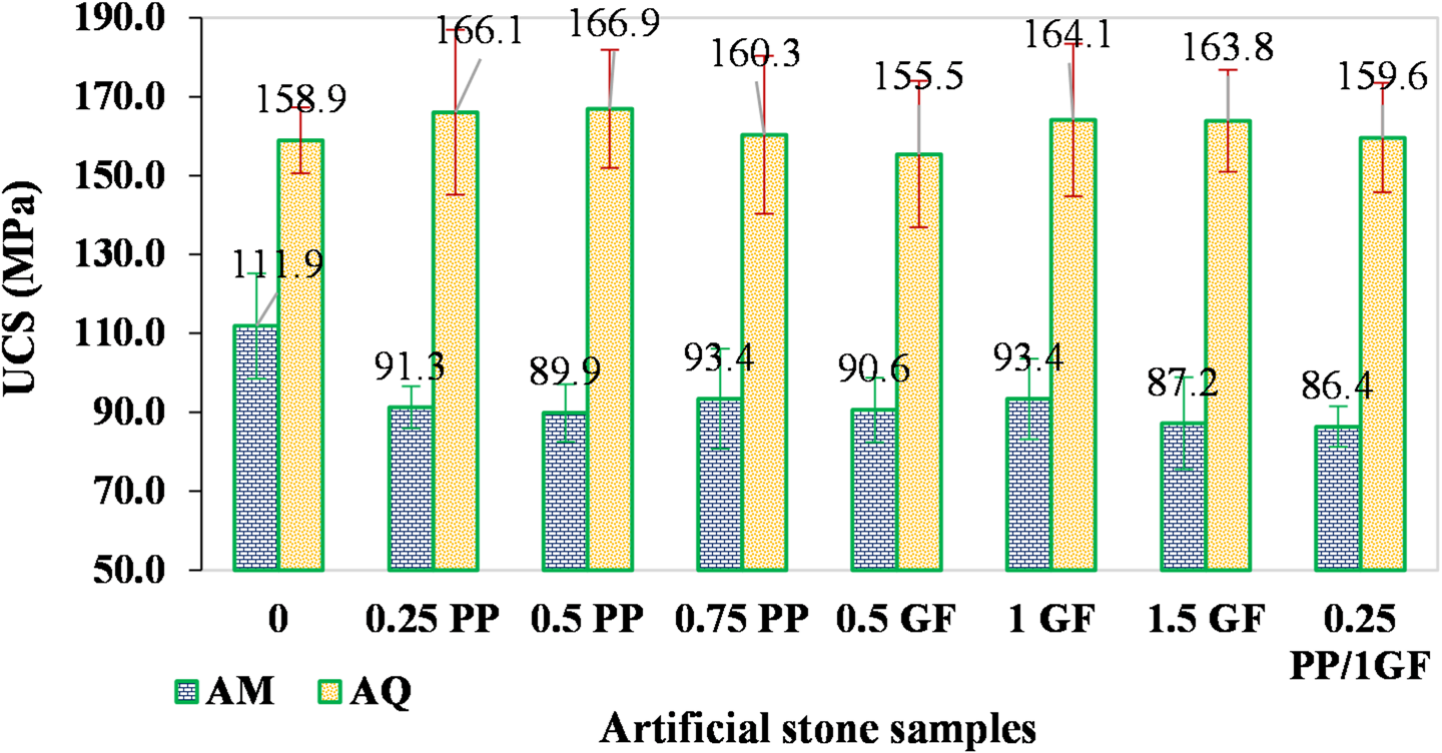
Variations of UCS in AM and AQ samples with varying contents of GF and PP fibers.

Figure 12 shows that UCS of AM samples generally decreased after fiber reinforcement compared to the fiber-free sample (AM-10R), dropping from approximately 111.9 MPa to below 94 MPa. The lowest strength was observed in the hybrid fiber sample (86.4 MPa). In contrast, the UCS of AQ samples (except for AQ-0.5G, which showed a slight decrease) increased slightly compared to the fiber-free sample (AQ-12R), rising from about 158.9 MPa to approximately 166 MPa in samples with 0.25% PP and 0.5% PP, and 164 MPa in samples with 1% G and 1.5% G (a maximum increase of about 5%).

**Fig. 13:**
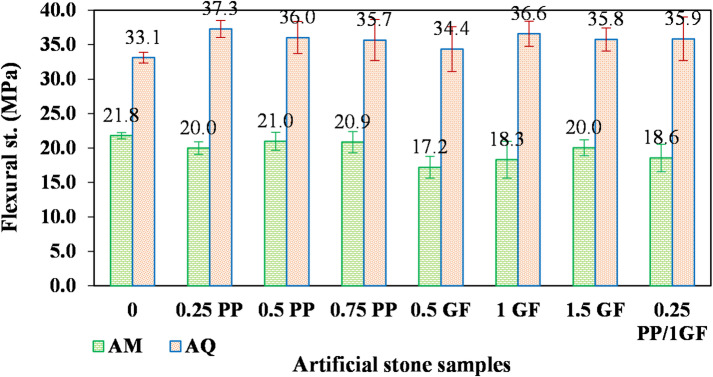
Variation of flexural strength (MPa) in AM and AQ samples with varying contents of GF and PP fibers.

The results demonstrate that the AMs and AQs exhibited divergent responses to fiber reinforcement. Similar trends were observed for their flexural strength (Fig. 13).

The flexural strength of the artificial marble decreased from 21.8 MPa in the fiber-free sample to a range of 17.2 to 21 MPa in the fiber-reinforced samples (Fig. 13). In contrast, the flexural strength of quartzite slightly increased after fiber incorporation, rising from 33.1 MPa to a range of 34.4–37.3 MPa (a maximum increase of approximately 12%).

## Discussion

The mechanical test results of fiber-reinforced samples are interpreted herein, along with explanations for their strength enhancement or reduction relative to fiber-free counterparts.

According to studies conducted by some researchers, the use of fibers has led to an increase in mechanical strength^19,53–55^, while others have reported a decrease in strength^22,23,56,57^.

In most studies, attempts have been made to attribute the reason for the increase or decrease in strength to the type, length, and ratio of fibers. However, when similar studies were reviewed, the reason for the decrease in strength was generally attributed to the excessive use of fibers, fiber agglomeration, and consequently, entrapped air^11^.

Arif Ulu et al. found that adding GF 0.15% to 1% by volume (vol%) to polymer concrete significantly reduced its mechanical strength. As the fiber content increased, the compressive strength dropped from 94.29 MPa (0% fibers) to 63.23 MPa (1% fibers), and the flexural strength decreased from 19.85 MPa to 13.07 MPa. SEM analysis revealed that higher fiber percentages led to increased fiber agglomeration, which hindered binder penetration and trapped more air bubbles, ultimately weakening the concrete^11^.

Kizilkanat et al. found that adding GF (0.25 to 1 vol%) to concrete had varying effects. While compressive strength experienced a modest 6% increase at 0.75 vol% fibers, flexural strength significantly improved by 32% at 0.5% vol% fibers, and tensile strength rose by 27% at 0.75vol% fibers. Beyond these optimal points, further fiber addition provided no additional benefits due to poor fiber distribution and agglomeration. A noted drawback was reduced workability, which was mitigated by adding a superplasticizer.

Based on the results obtained from the production of fiber-free samples in this research, it was determined that the quality and compressive strength of the artificial stone (with the same aggregate and resin type) are highly dependent on the total porosity. As the porosity increases, the compressive strength of the stone decreases significantly.

**Fig. 14:**
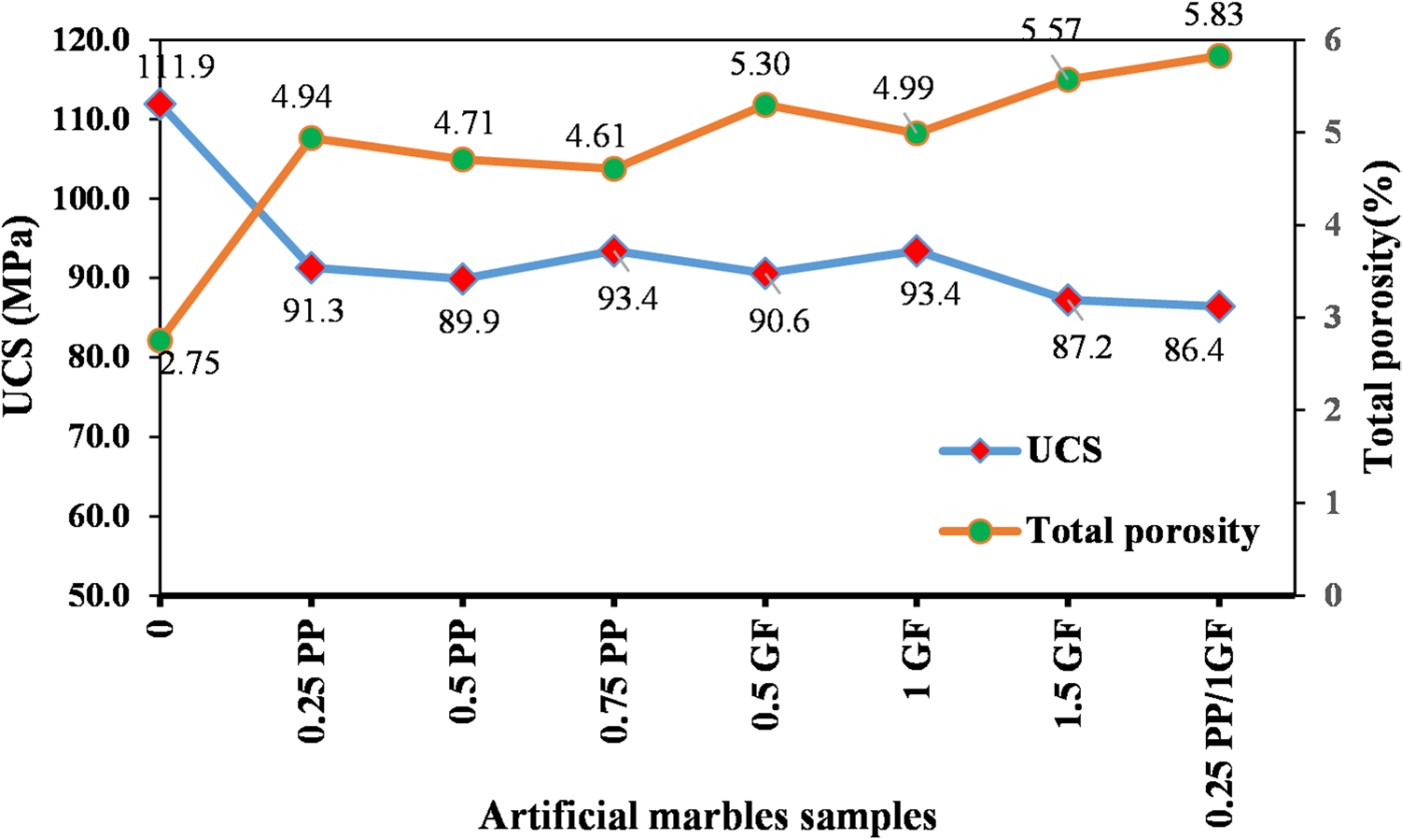
Comparison of UCS and total porosity trends in fiber-reinforced AMs.

This study depicted that incorporating fibers into the artificial marble significantly increased its total porosity. To further investigate this correlation, Fig. 14 plots both the total porosity and compressive strength values of the fiber-reinforced samples. The curves demonstrate a nearly inverse relationship, confirming that the increase in porosity directly contributes to the reduction in compressive strength. Specifically, the porosity rose by 1.8% to 3% as a result of fiber incorporation.

**Fig. 15:**
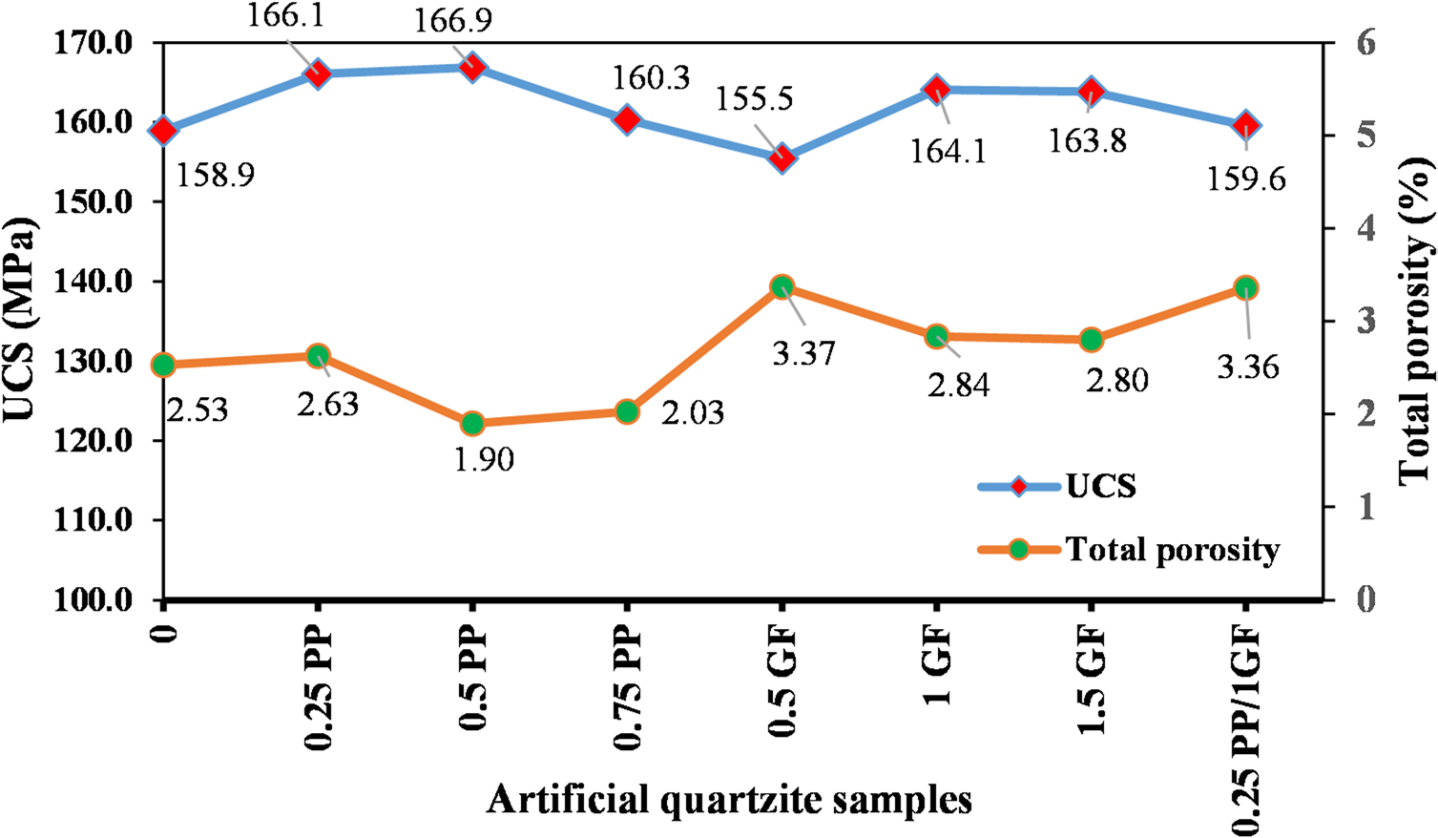
Comparison of UCS and total porosity trends in fiber-reinforced AQs.

The addition of fibers to the artificial marble paste (containing fine powder, aggregate, and resin) significantly reduced fluidity and workability, resulting in a stiffer mixture. This impaired vibration efficiency during compaction, limiting air evacuation and ultimately increasing porosity while reducing strength.

The total porosity and compressive strength of fiber-reinforced artificial quartzite samples are plotted in Fig. 15. According to the figure, the total porosity of artificial quartzite did not increase significantly with the addition of fibers and even decreased in two cases (0.5PP and 0.75PP). The artificial quartzite paste maintained adequate workability and fluidity even after fiber incorporation, allowing efficient entrapped air removal through vibration, vacuum, and compression. The maximum porosity increase in artificial quartzite after fiber incorporation does not reach 1%. Specifically, according to the curves in Fig. 15, when the porosity increase is less than 0.5%, the fibers fulfill their role and slightly enhance the strength of the resin-based artificial stone. However, strength degradation commences once the porosity increase surpasses 0.5%. This is exemplified by sample 0.5 GF (artificial quartzite with 0.5% GF), where a 0.9% rise in porosity resulted in an approximate 2% loss in UCS relative to the fiber-free sample.

### Statistical analysis

To evaluate the statistical significance of the observed variations in mechanical properties among different composite formulations, a one-way Analysis of Variance (ANOVA) was performed. ANOVA is a statistical method used to determine whether the means of multiple groups are significantly different from each other, considering both within-group and between-group variances.

**Table 6:** Statistical analysis of mechanical properties in artificial Stones.

Property type	Material type	F-value	df	*p*-value	Significance
Compressive strength	Artificial marble	4.19	7,40	0.0015	**
	Artificial quartzite	0.22	7,36	0.976	NS
Flexural strength	Artificial marble	6.41	7,31	< 0.001	**
	Artificial quartzite	2.33	7,28	0.054	NS

Based on the results (Table 6) of one-way analysis of variance (ANOVA), the compressive strength of fiber-reinforced artificial marble samples showed a significant reduction (F(7,40) = 4.19, *p* = 0.0015). This strength reduction was directly correlated with increased porosity in these samples, as regression analysis revealed a strong inverse relationship between these parameters, according to Eq. (3):

3 $$UCS = - 8.26 \times {\text{n\% }} + 133.01$$

where UCS (MPa), n%: Total porosity.

**Fig. 16:**
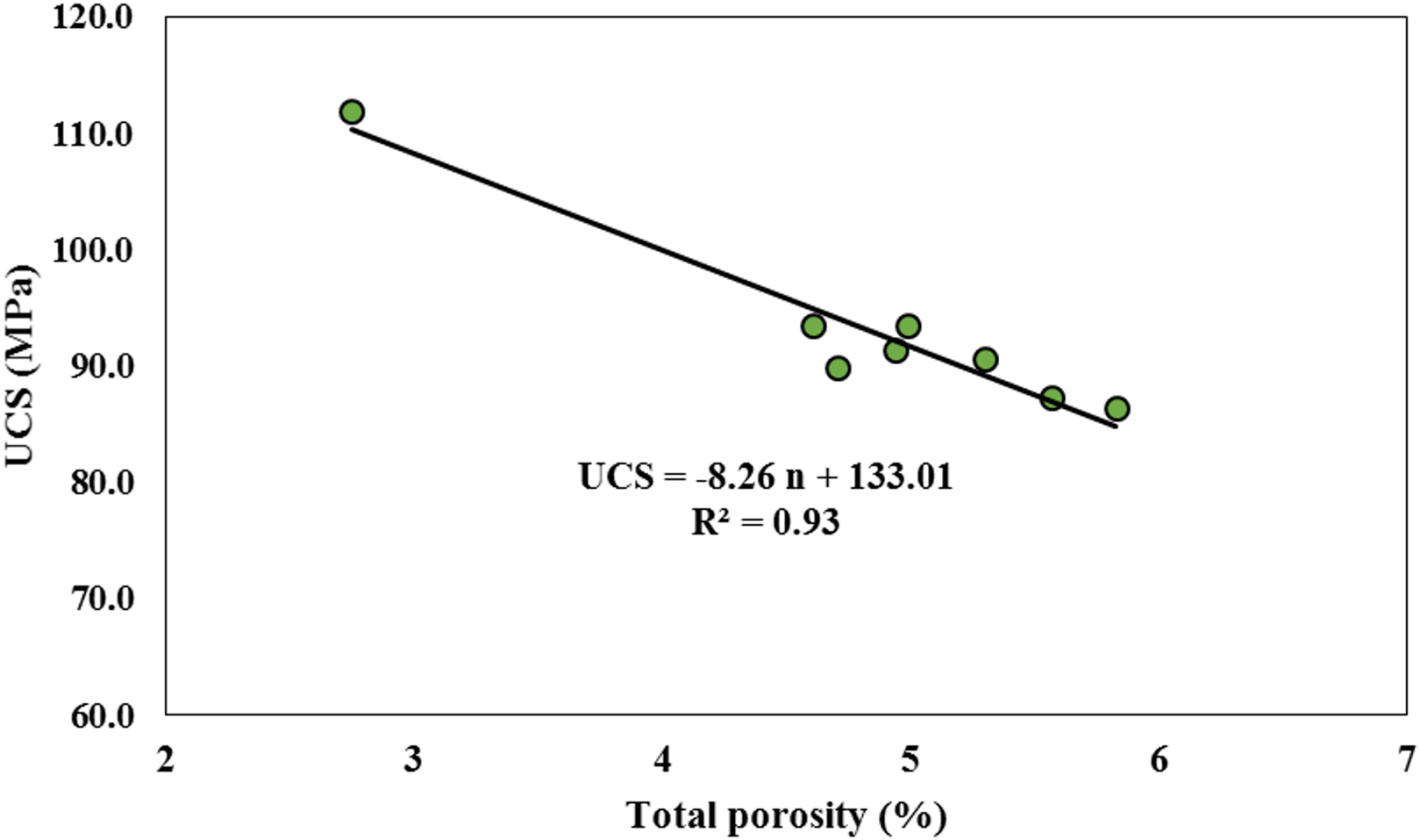
Relationship between UCS and total porosity in artificial marble (AM) samples.

According to this relationship, each 1% increase in porosity reduces compressive strength by approximately 8.26 MPa (Fig. 16). In contrast, fiber-reinforced artificial quartzite maintained its compressive strength across all formulations with no significant differences (F(7,36) = 0.22, *p* = 0.976). Fiber reinforcement significantly reduced flexural strength in artificial marble (F(7, 31) = 6.41, *p* < 0.001), with all fiber types (PP, GF, hybrid) showing lower values than the control. In contrast, artificial quartzite showed no significant reduction (F(7, 28) = 2.33, *p* = 0.054), the p-value approached significance, indicating a trend toward improvement with fiber addition, particularly at low PP doses (up to + 12% in AQ-0.25PP).

In summary, the increased porosity in fiber-reinforced artificial marble was the main factor responsible for the reduction in compressive strength, whereas in artificial quartzite, fibers exerted a more effective influence on increasing flexural strength due to minimal changes in porosity levels.

From a micromechanical viewpoint, the enhanced flexural performance in quartzite stems from effective fiber crack-bridging, which is critical under flexural loading where tensile stresses prevail; in compression, however, matrix crushing and shear dominate failure, limiting fiber contributions to strength.

### The main reason of divergent behavior in artificial marble and quartzite with fiber

Despite the same production method (applying the same vacuum, vibration and compression), the same grain size of sand particles, and the type of resin, it seemed that the different behavior of artificial quartzite and marble with the addition of fibers was due to the difference in fineness between silica and carbonate rock powder.

**Fig. 17:**
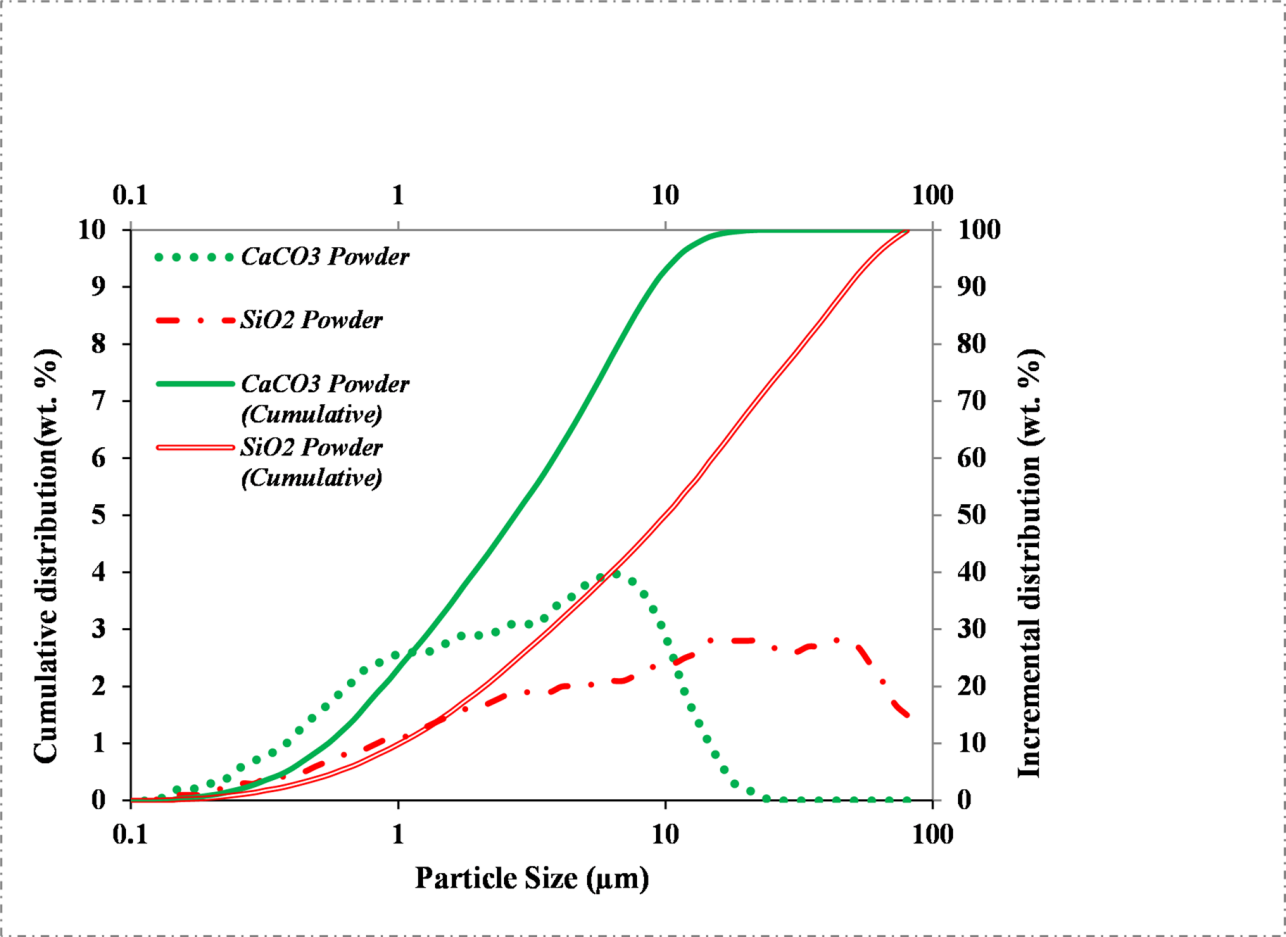
Particle size distribution of calcium carbonate and silica fine powders measured by laser diffraction granulometry.

To characterize the exact particle size distribution of the fine powders (all passing a No. 200 sieve), laser diffraction analysis was conducted. The results, shown in Fig. 17, demonstrate that the carbonate powder had a finer particle size distribution (1–11 μm) than the silica-based powder (2–80 μm). Due to its lower hardness and mechanical strength relative to quartzite, marble produces finer powder when milled under constant energy conditions.

The increased fineness of the carbonate powder resulted in a larger specific surface area, which enhanced resin adhesion but concurrently reduced the paste’s workability. Consequently, under identical processing conditions, the evacuation of air from the mixture of resin, ultra-fine powder, and intertwined fibers was less effective, which resulted in a product with higher total porosity in fiber-reinforced artificial marbles.

#### SEM investigation and comparison of artificial stone’s microstructure

The microstructure of the artificial stone specimens was characterized in detail using SEM). This examination evaluated critical structural attributes including the aggregate-matrix interfacial zone, distribution of stone powder within void spaces, micro-crack formation, pore structure, and fiber dispersion patterns.

**Fig. 18:**
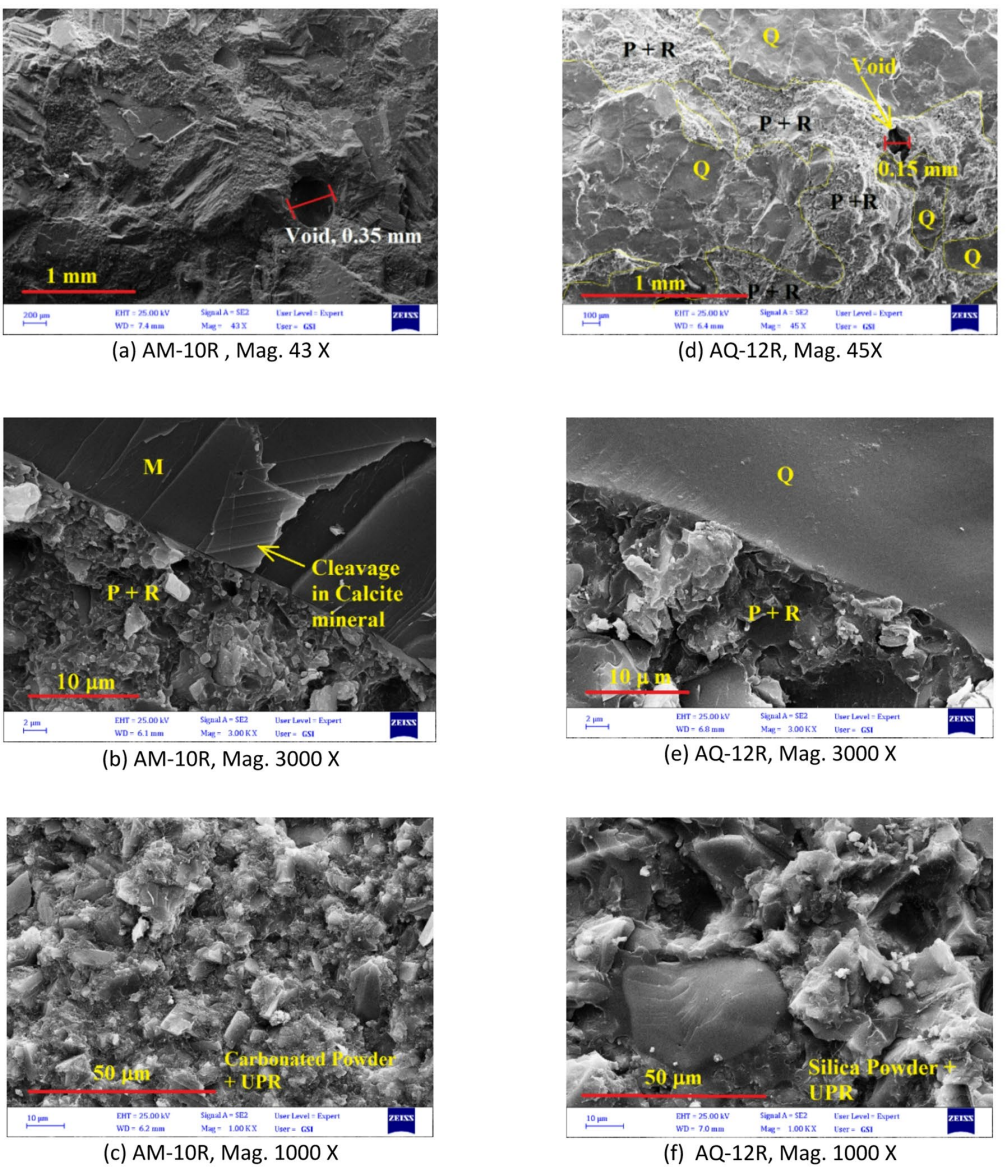
SEM images of fiber-free artificial stones: (a–c) AM-10R (marble-based) and (d–f) AQ-12R (quartzite-based). (M: Marble, Q: Quartzite, P + R: Powder + Resin as labeled in the images).

The investigation commenced with SEM imaging of two fiber-free reference samples (AM-10R and AQ-12R), followed by a comparative examination of fiber-reinforced counterparts. As shown in Fig. 18a, d, the interstitial spaces between coarse aggregates in both samples are adequately filled by the resin-powder matrix. However, each matrix exhibits fine bubbles, measuring 0.35 mm and 0.15 mm in diameter, In the SEM images and the reported pore sizes represent manual measurements of characteristic large pores, provided to qualitatively illustrate microstructural features influencing mechanical behavior. The volumetric percentage of these pores has been quantitatively captured through total porosity measurements.

Consequently, despite the simultaneous application of VVC method during production (which results in samples with high mechanical strength), these scattered pores persist. Optimizing these processing parameters to minimize such porosity would be a technical concern for authors in future studies.

Figure 18b, e, demonstrates effective adhesion and wetting, with no gaps observed between the marble (M) and quartz (Q) aggregates with the resin-powder mixture (P + R). This strong interfacial bonding is a key factor in the high mechanical strength of the materials.

Figure 18c, f, presents the resin-stone powder matrix at 1000x magnification. Laser particle size analysis confirms the carbonate (marble) powder is finer than the silica (quartzite) powder. Although this fineness enhances resin-carbonate adhesion, it concurrently reduces the workability and vibrational compactability of the fiber-reinforced marble paste. This ultimately led to higher porosity and diminished strength in these specimens.

**Fig. 19:**
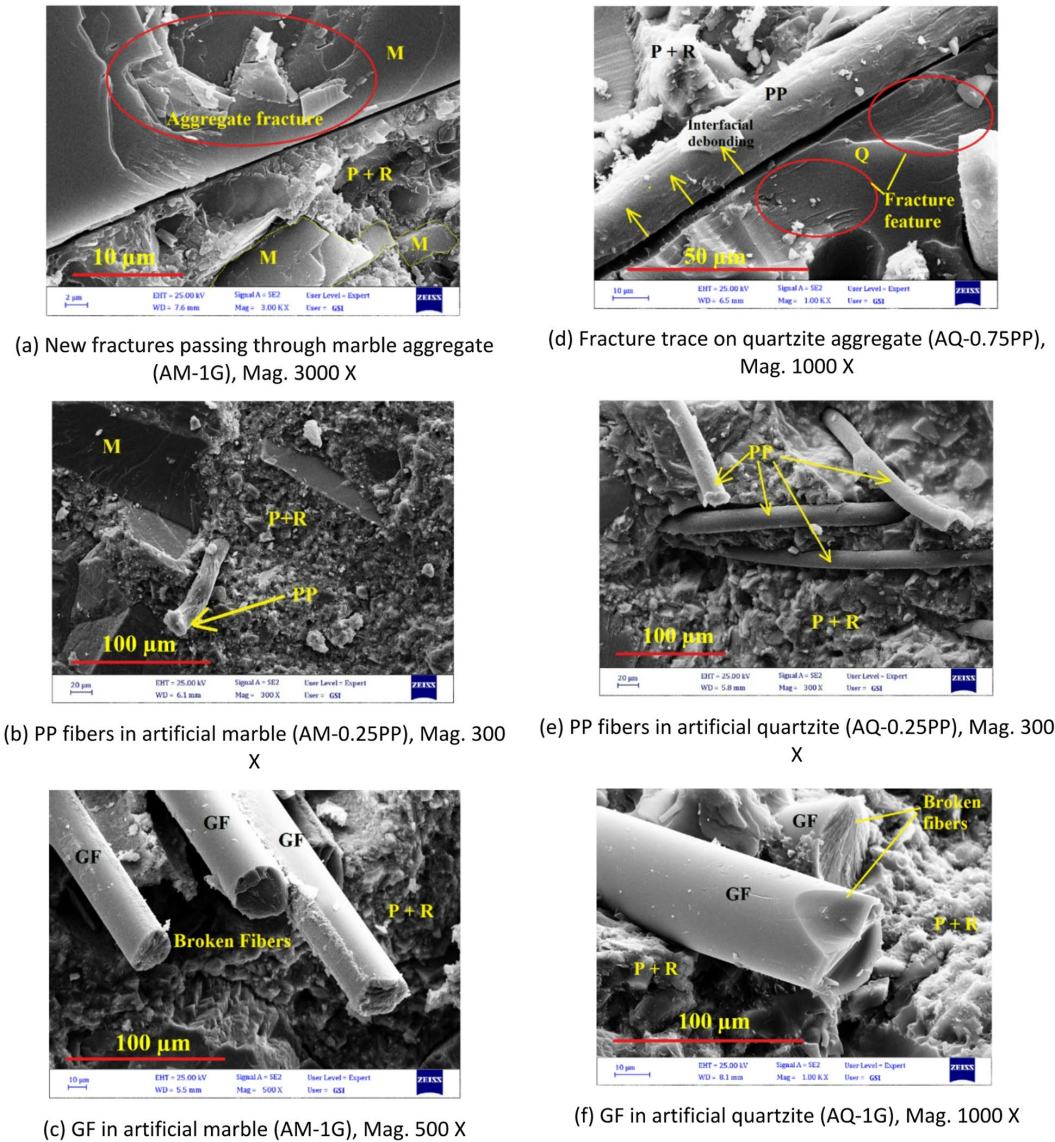
SEM micrographs of fiber-reinforced artificial stones: (**a**–**c**) artificial marble and (**d**–**f**) artificial quartzite. (M: Marble, Q: Quartzite, P + R: Powder + Resin as labeled in the images).

Microstructural observations of fiber-reinforced artificial stone mixtures are presented in Fig. 19a–e, highlighting several key findings:

According to Fig. 19a, b, the fracture planes after the UCS test even passed through the quartzite and marble aggregates. This finding is highly significant and clearly demonstrates that, in addition to the resin, the aggregate type plays a critical role in the strength of the artificial stone. The use of harder aggregates directly enhances its strength that quantitatively confirmed by mechanical tests in this study.

Based on Fig. 19b, c, after fracture in UCS test, clear tearing of PP fibers and fracture of GF are observed respectively. The same behavior is seen in artificial quartzite samples (Fig. 19e, f). This indicates that both fibers contributed to load-bearing, and the resin-powder matrix adhered well to the fibers, transferring stress to them until failure.

The interfacial adhesion between fibers and the unsaturated polyester resin (UPR) depends strongly on chemical compatibility^58^. Glass fibers (GF) contain silanol groups (Si–OH) on their surface, which can form covalent bonds with the polyester matrix through the silane coupling agent, resulting in strong interfacial bonding and efficient stress transfer^59,60^. In contrast, polypropylene (PP) fibers are nonpolar and chemically inert; thus, their adhesion with UPR is mainly mechanical rather than chemical^61^. Nevertheless, the improved strength observed in both PP- and GF-reinforced quartzite composites suggests that physical mechanisms such as fiber bridging and crack deflection also contributed effectively to reinforcement^59^. Future studies may explore alternative resin systems such as epoxy or vinyl ester, which possess higher polarity and may enhance fiber–matrix bonding efficiency.

While fiber incorporation may increase porosity, optimizing production parameters (vacuum, vibration) can mitigate this effect and enhance stone strength. This study revealed that fiber incorporation reduced strength in artificial marbles due to significant porosity increase, whereas in quartz-based samples, it enhanced flexural strength by approximately 12% as porosity did not change significantly.

**Fig. 20:**
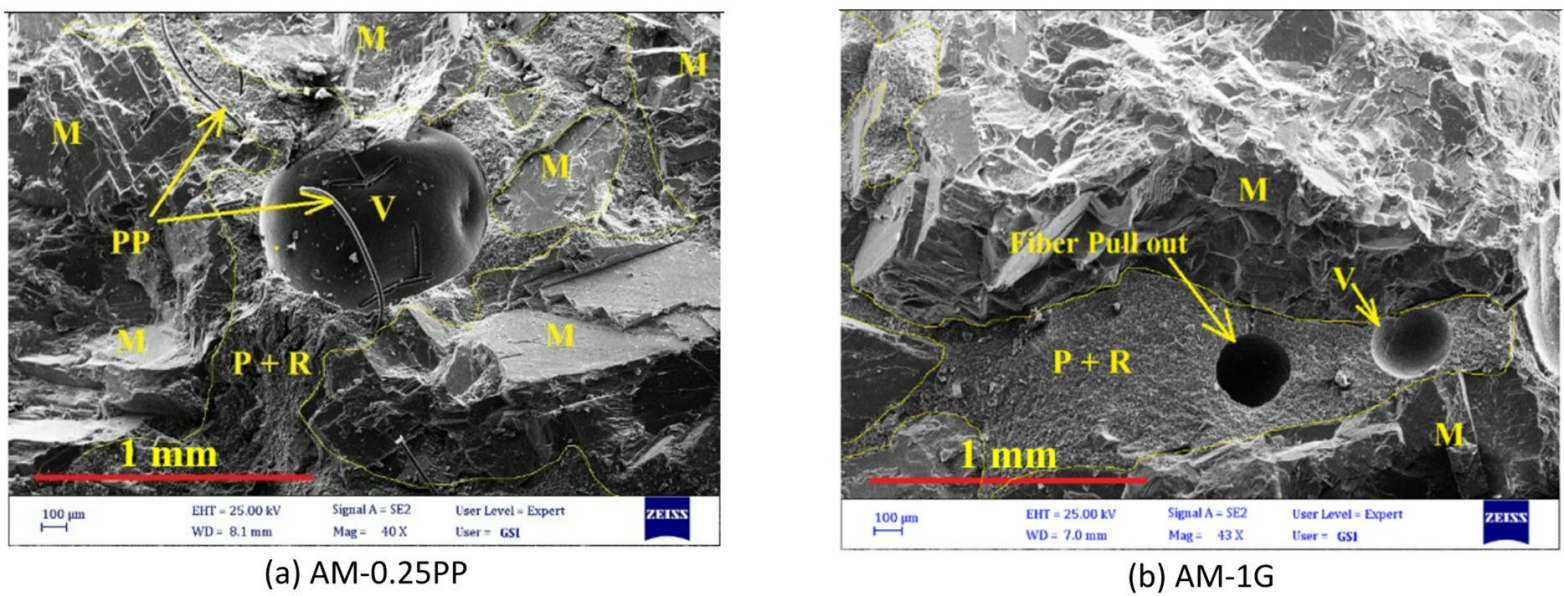
SEM micrograph showing increased porosity in the fiber-reinforced artificial marble matrix (M: Marble, P + R: Powder + Resin, V: Void).

**Fig. 21:**
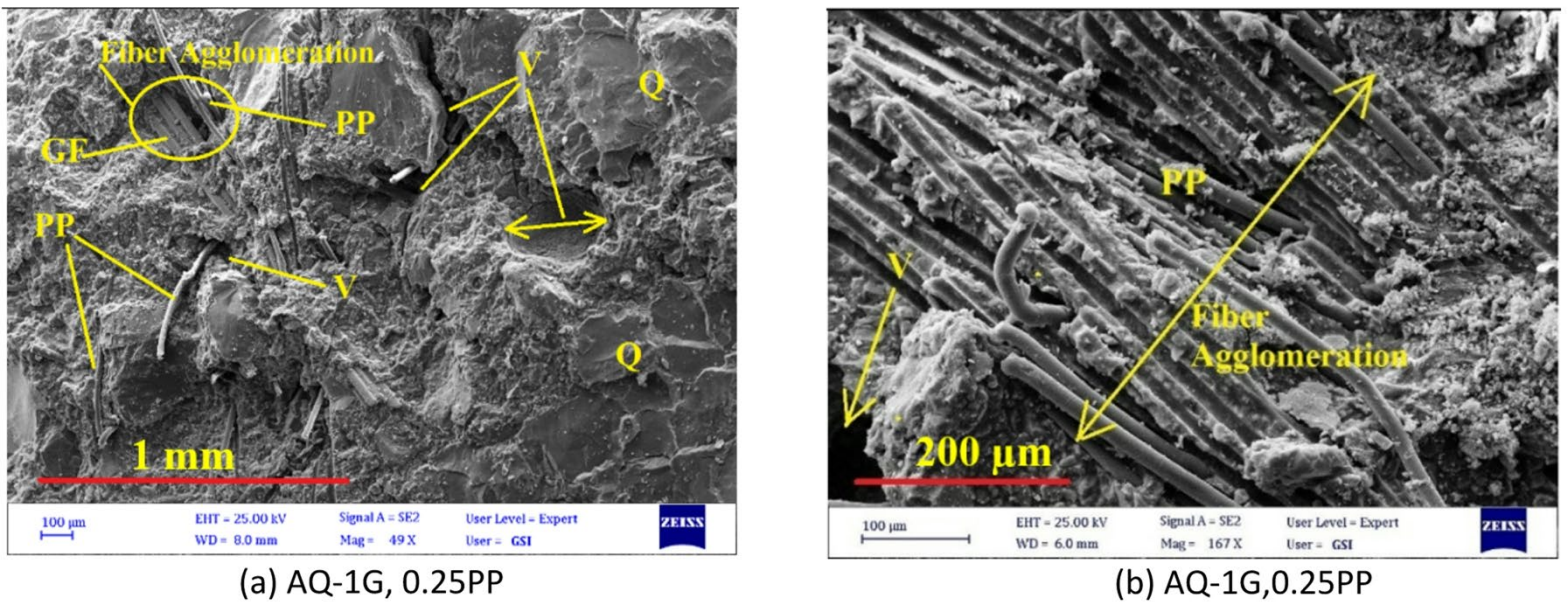
Fiber agglomeration and increased porosity in hybrid fiber samples (Q: Quartzite, P + R: Powder + Resin, V: Void).

Therefore, when incorporating fibers into artificial stone, it is crucial to mitigate their tendency to increase porosity by optimizing key production parameters. Figure 20 shows that one of the challenges of using fibers in resin-based stone could be the increase in porosity or the rise in the number and size of trapped pores within the matrix. As demonstrated in the SEM image (Fig. 21), the use of hybrid GF-PP fibers has led to increased porosity due to issues such as poor fiber distribution and agglomeration (as seen in Fig. 21a and b), resulting in reduced mechanical strength. Thus, the use of a hybrid GF-PP fiber combination in resin based artificial stone is explicitly not recommended.

## Conclusions

In this study, two different types of aggregates were deliberately used in the production of resin-based artificial stones to evaluate not only the effect of fiber reinforcement but also the influence of aggregate type on the physical-mechanical properties of the composites. The results primarily revealed that the use of stronger aggregates leads to the production of artificial stones with both higher UCS (Unconfined Compressive Strength) and higher flexural strength.

Subsequently, the main phase of the study focused on examining the effect of incorporating fibers, specifically glass fiber (GF) and polypropylene (PP) fiber, into these artificial stones. The results demonstrate that the effectiveness of fiber reinforcement is contingent upon its impact on total porosity. Fibers only enhance strength when they do not increase the total void content within the material.

Despite identical manufacturing processes, the addition of fibers yielded contrasting results between the two stone types: the compressive and flexural strength of artificial marble decreased, whereas the strength of artificial quartzite improved in most samples. This divergence was attributed to porosity changes; tests showed that fiber incorporation generally increased porosity and reduced strength in marble composites, while the porosity of quartzite composites remained largely unaffected. Further analysis identified a key material difference: the particle size distribution of the fillers. The marble powder filler was significantly finer (1–11 μm) than the quartzite powder (2–80 μm), a critical factor influencing the composite’s behavior during manufacturing and its final properties. This higher fineness significantly increased the specific surface area of the carbonate powder, leading to greater resin adhesion and reduced workability in the artificial stone paste. Under identical vacuum, vibration, and compression conditions, air evacuation from the adhesive matrix of the ultra-fine carbonate powder and resin, combined with intertwined fibers becomes more difficult leading to higher total porosity and lower strength in fiber-reinforced artificial marbles. Thus, it can be concluded that with the addition of fibers, manufacturing parameters (vacuum, vibration, and compression) must be increased to facilitate air removal, allowing the fibers to fulfill their role and perform effectively. Future studies are recommended to focus on optimizing VVC parameters for large-scale industrial production and assessing long-term durability under realistic exposure conditions (moisture, temperature variations, UV radiation, and chemical attack) to support industrial adoption in engineered stone applications.

## Supplementary Information

Below is the link to the electronic supplementary material.

Supplementary Material 1

## Data Availability

All data generated or analyzed during this study are included in this published article.
